# Corelease of Genotoxic Polycyclic Aromatic Hydrocarbons and Nanoparticles from a Commercial Aircraft Jet Engine – Dependence on Fuel and Thrust

**DOI:** 10.1021/acs.est.3c08152

**Published:** 2024-01-11

**Authors:** Norbert V. Heeb, Maria Muñoz, Regula Haag, Simon Wyss, David Schönenberger, Lukas Durdina, Miriam Elser, Frithjof Siegerist, Joachim Mohn, Benjamin T. Brem

**Affiliations:** † Empa, Swiss Federal Laboratories for Materials Science and Technology, Laboratory for Advanced Analytical Technologies, Überlandstrasse 129, CH-8600 Dübendorf, Switzerland; ‡ Empa, Swiss Federal Laboratories for Materials Science and Technology, Laboratory for Air Pollution/Environmental Technology, Überlandstrasse 129, CH-8600 Dübendorf, Switzerland; § Empa, Swiss Federal Laboratories for Materials Science and Technology, Automotive Powertrain Technologies Laboratory, Überlandstrasse 129, CH-8600 Dübendorf, Switzerland; ∥ SR Technics Switzerland AG, Zurich-Airport, CH-8058 Kloten, Switzerland

**Keywords:** genotoxic polycyclic aromatic hydrocarbons (PAHs), hydro-processed esters and fatty acids (HEFA), Trojan horse effect, particle number (PN), ultrafine particles

## Abstract

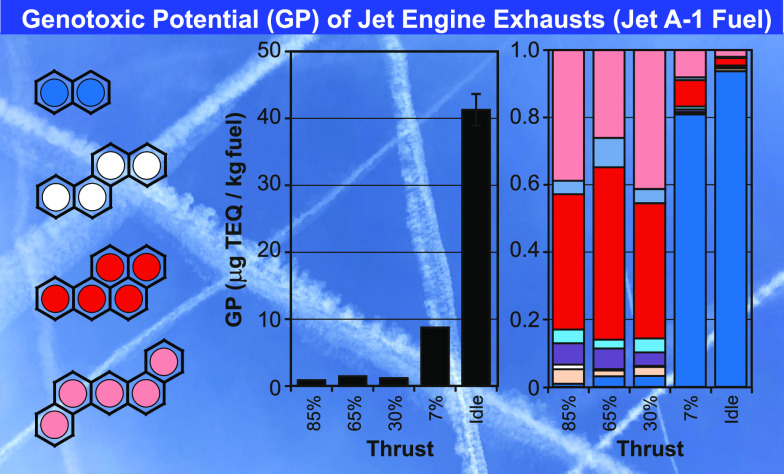

Jet engines are important contributors to global CO_2_ emissions and release enormous numbers of ultrafine particles into different layers of the atmosphere. As a result, aviation emissions are affecting atmospheric chemistry and promote contrail and cloud formation with impacts on earth’s radiative balance and climate. Furthermore, the corelease of nanoparticles together with carcinogenic polycyclic aromatic hydrocarbons (PAHs) affects air quality at airports. We studied exhausts of a widely used turbofan engine (CFM56–7B26) operated at five static thrust levels (idle, 7, 30, 65, and 85%) with conventional Jet A-1 fuel and a biofuel blend composed of hydro-processed esters and fatty acids (HEFA). The particles released, the chemical composition of condensable material, and the genotoxic potential of these exhausts were studied. At ground operation, particle number emissions of 3.5 and 0.5 × 10^14^ particles/kg fuel were observed with highest genotoxic potentials of 41300 and 8800 ng toxicity equivalents (TEQ)/kg fuel at idle and 7% thrust, respectively. Blending jet fuel with HEFA lowered PAH and particle emissions by 7–34% and 65–67% at idle and 7% thrust, respectively, indicating that the use of paraffin-rich biofuels is an effective measure to reduce the exposure of airport personnel to nanoparticles coated with genotoxic PAHs (Trojan horse effect).

## Abstract 2

The inhalation of jet engine exhausts including genotoxic PAHs and persistent soot nanoparticles (Trojan horse effect) is a health threat for exposed personnel and passengers, which can be reduced by using paraffin-rich biofuels.

## Introduction

### Implementation of Particle Number-Based Legislation for Jet Engines

In an expanding market with passenger growth rates that may double up to 2050, the aviation sector is facing several challenges, such as reducing emissions affecting climate and human health and the implementation of sustainable aviation fuels. Up to now, combustion of fuels in jet engines has been optimized for performance, stability, and safety. However, also global standards limiting gaseous emissions of carbon monoxide (CO), hydrocarbons (HC, as CH_4_-equivalents), and nitrogen oxides (NO_*x*_, as NO_2_-equivalents) have been established.^1^ Up to 2020, particulate matter (PM) emissions were regulated as the smoke number. In 2019, new emission standards for nonvolatile particulate matter mass (nvPM) and number (nvPN) have been introduced.^2−4^ These additional metrics reflect other more health and environmental related properties of combustion exhausts. Before setting new PN-based emission limits, robust and reproducible sampling protocols had to be established, which include appropriate dilution devices and particle counting instrumentation.^5−8^

### Transport of Genotoxic Adsorbates by Nanoparticles: The Trojan Horse Effect

Exhausts of combustion engine vehicles contain billions of primary particles/ccm. These spherical particles with diameters of 10–15 nm quickly form branched agglomerates with mean geometric diameters of 70–90 nm.^9^ High PN emissions are also found for heavy-duty diesel engines applied in construction machinery and mining equipment.^10^ In other words, miners and construction workers exposed to nonfiltered exhausts inevitably inhale large numbers of diesel particles. Based on findings of an occupational health study on 12315 miners, it was found that the exposure to nontreated diesel exhaust was responsible for 198 lung cancer death (>16000 deaths per million) in the examined cohort.^11^ Reports on the same cohort 18 years later with now 409 lung cancer death (>33000 deaths per million) confirmed the carcinogenicity of diesel exhaust.^12^ In 2012, in response to the U.S. miners’ study and other evidence, the World Health Organization (WHO) has announced that the exposure of humans to nontreated diesel exhaust induces lung cancer.^13,14^ The health risks of the inhalation of nanoparticles carrying carcinogenic or mutagenic compounds cannot be underestimated, considering recent findings in the field of nanotoxicology.^15^ It was shown that persistent, nonsoluble nanoparticles <100 nm can penetrate the alveolar membrane of the human lung reaching the blood circulation system and with it every organ supported by blood.^16,17^ Furthermore, sub-100 nm particles can also penetrate the placenta membrane and are transferred from the mother to the fetal blood circulation system.^18^ Thus, the inhalation of nondegradable nanoparticles, which transport genotoxic material in the human body like a Trojan horse, represents a severe health threat. Considering the similarities of diesel and jet engine particles, it is one goal of this study to also evaluate the genotoxic potential of jet engine exhausts.

### PN-Based Legislation, a Key Factor to Implement Particle Emission Control Technologies

A PN-based legislation has triggered the search for emission control technologies to lower particle emissions of combustion engines. Particle filters are now the most efficient technology to remove soot and ash particles from exhausts of diesel and gasoline engines.^10^ They also lower emissions of genotoxic material adsorbed on such particles.^19−22^ Already since 1998, diesel machinery (>47 kW) for tunnel construction has to be equipped with certified particle filters in Switzerland^10^ and PN-based emission regulations for construction machinery were introduced to the Swiss Clean Air Act in 2009.^23^ In 2008, a first PN-limit of 6 × 10^11^ particles/km was introduced for diesel passenger cars and light-duty vehicles.^24^ This forced the implementation of particle filters and low-sulfur fuels. Already in the year 2000, Peugeot introduced first in-series DPF-vehicles.^25^ In 2012, a first PN-limit of 6 × 10^12^ particles/km was introduced for gasoline-direct injection (GDI) vehicles in the EU, which was further lowered to 6 × 10^11^ particles/km in 2018.^26^ It was found that Euro-3 to Euro-6 GDI-vehicles, used as reference herein, release on average above 6 × 10^11^ particles/km.^27^

For obvious reasons, particle filters are not an option for jet engines. The optimization of combustion conditions and the use of better fuels remain strategies to lower jet engine particle emissions. Fuels with oxygen-containing compounds like ethanol reduce nanoparticle and genotoxic PAH emissions of GDI vehicles but are not allowed in commercial aviation.^28^ However, improved fuel formulations are an interesting option for the aviation industry too. Jet fuels produced from renewable sources will help to lower life cycle CO_2_ emissions. Fuels with high paraffin and low aromatics contents can lower jet engine particle emissions.^29−31^ In this respect, hydro-processed esters and fatty acids (HEFA), which are considered as biofuels, and synthetic fuels, obtained from Fischer–Tropsch synthesis, gained importance in aviation.^32,33^ Paraffin-rich jet A-1 fuel has a high energy density of 43 MJ/kg (this work), allowing long-distance flights, where fuel weight is a limiting factor, whereas oxygen-containing fuels like methanol (16 MJ/kg), ethanol (25 MJ/kg), and fatty acid methyl-esters (FAME, 37 MJ/kg), widely used as biofuels for vehicles, have considerably lower energy densities.

During activities to establish a PN-based legislation for aircraft engines, a sampling system was developed, compliant with the International Civil Aviation Organization (ICAO Annex 16 Vol. II), to characterize jet engine particles for their mass and number emissions, size and optical properties.^34−38^ We hypothesized that jet engine particles are numerous and small, offering sufficient surface to carry genotoxic adsorbates (Trojan horse effect) and collected complete jet engine exhausts including solid, condensable, and gaseous compounds at different thrust levels. We used the platform at SR-technics to (i) investigate nonregulated emissions of jet engines, (ii) to evaluate the impact of fuels and engine thrust on the chemical composition of exhausts, and (iii) to assess the genotoxic potential of jet engine exhausts in comparison to other combustion engines.

## Methodology

### Jet Engine, Test Cycle, and Fuels

A well run-in turbofan engine (CFM56–7B26) was operated first at 85 and then at 65, 30, and 7% sea level thrust and at idle. According to ICAO emission certification procedures, a specific combustor inlet temperature (T3) was used to control the engine at each test point. The test points chosen were based on a correlation between engine thrust and T3 at sea level determined from a calibrated engine performance model for this engine type. Engine conditions were equilibrated and kept constant for 60 min during exhaust sampling. This corresponded to an overall fuel consumption of 7800 and 8200 kg/cycle for Jet A-1 fuel and the HEFA blend under the specific ambient conditions 2 and 17 °C, respectively. Figure S1 displays the test cycle, exhaust gas temperatures, and fuel consumption at different thrust levels when using Jet A-1 fuel and the HEFA blend.

Tables S1 and S2 report the characteristics of the fuels used in this study. Jet A-1 fuel, taken from Zürich Airport, complied with the Appendix 4 to the ICAO Environmental Technical Manual, Volume II specifications. HEFA was imported from SkyNRG (Amsterdam, The Netherlands) and blended (32%v) with Jet A-1 fuel (68%v).^39^ The Jet A-1 fuel and the HEFA blend had aromatic contents of 17.8% and 11.3% and smoke points of 21.9 and 23.0 mm, respectively. The latter indicates that a reduced soot formation is expected for the HEFA blend, as previously shown.^32^ Both fuels fulfilled the specification for jet engine operation. However, pure HEFA, with a smoke point >40 mm and an aromatic content <0.5%, would not fulfill the current jet fuel specifications. Nevertheless, HEFA blends up to 50% have been used in jet engines and are now considered as sustainable aviation fuels by the ICAO.

### Testing Facility, Exhaust Sampling, and Analyses

**Figure 1:**
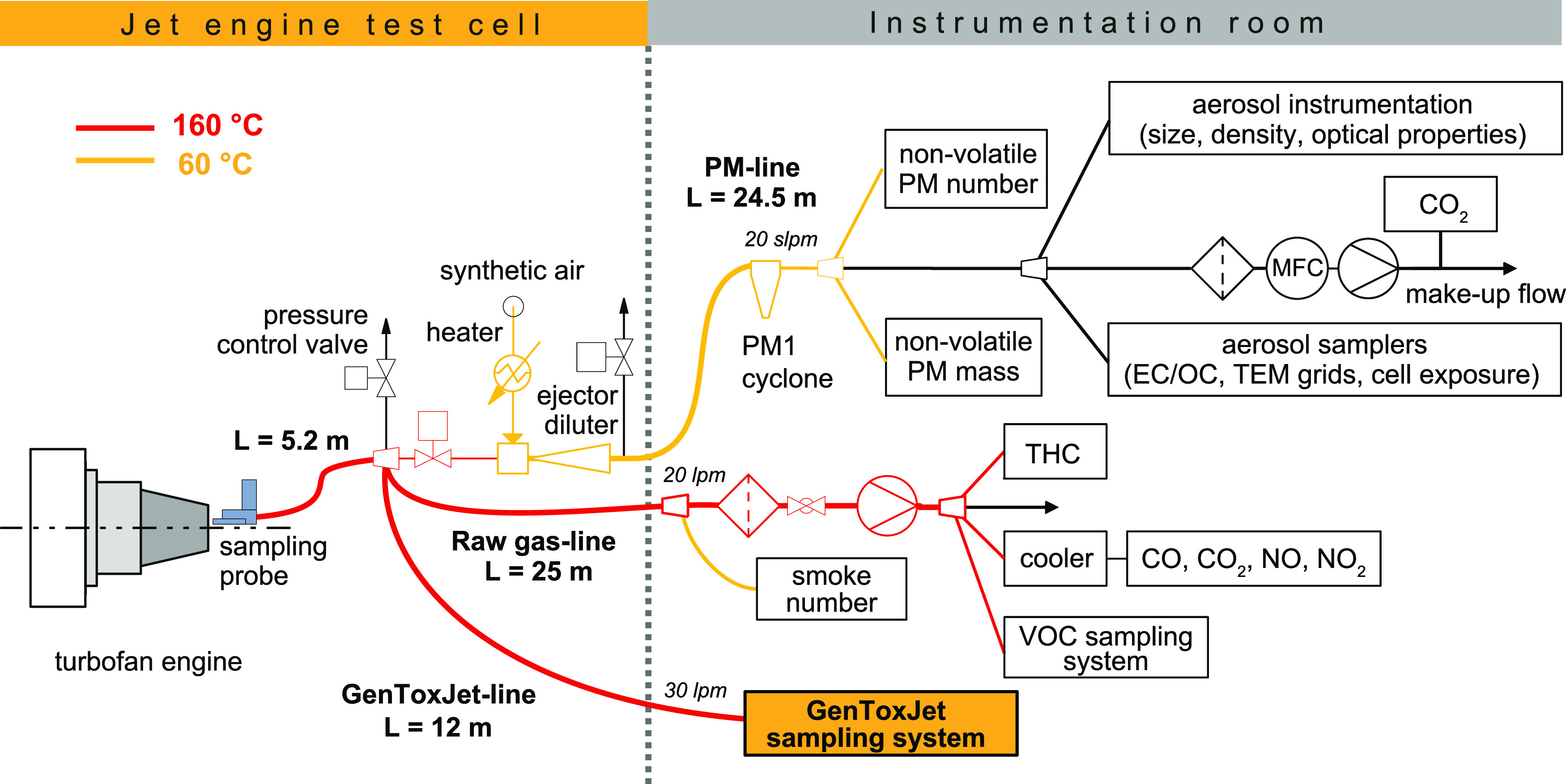
Experimental setup at the SR-Technics facility at Zurich airport, Switzerland. The turbofan engine (CFM56–7B26) was operated in the test cell following a five-stage cycle at 85, 65, 30, 7% thrust, and idle. Exhaust samples were collected through a sampling probe (L = 5.2m) and split into three lines. The PM-line (L = 24.5m), fed with exhaust and 10-fold diluted with synthetic air, was kept at 60 °C and used to measure nonvolatile particulate matter mass and number (nvPM, nvPN). The raw gas line (L = 25m), kept at 160 °C, was used to monitor major exhaust constituents (CO_2_, CO, NO, NO_2_, THC) and the smoke number. The GenToxJet-line (L = 12m), kept at 160 °C, was used to sample undiluted exhausts containing solid, condensable, and gaseous pollutants. The all-glass sampling device is described separately.

A scheme of the sampling devices and monitors used to collect, dilute, and examine jet engine exhausts for the major exhaust constituents CO_2_, CO, NO, NO_2_, and THC is shown in Figure 1. The system was installed at the SR-technics testing facility at Zurich Airport, Switzerland.^39^

The setup included a comprehensive battery of instruments to sample, dilute and to monitor nonvolatile particle emissions (PM-line, L = 24.5 m, 60 °C).^39^ This setup has been validated against other PM- and PN-instruments and has become the new standard procedure for nvPN and nvPM measurements approved by ICAO. A single point sampling probe was used at a predetermined location that is representative in terms of a carbon balance of the emissions and engine fuel consumption. This probe has been extensively used in previous campaigns. More details on particle measurements with this approach can be found elsewhere.^35,36,39,40^

Figure 1 also includes the GenToxJet-line (L = 12m, 160 °C). Details on the glass devices used to collect solid, condensable, and gaseous compounds at ∼0 °C are shown in Figure S2. The glass apparatus included a filter packed with glass wool, a condenser, two wash bottles in a cooling bath (0 °C), and a two-stage XAD-resin bed.^41^ The filter/condenser method is described in the European standard EN-1948–1.^41^ The glass devices were precleaned and baked out in an oven (450 °C) and assembled and disassembled on site. Aliquots of ^13^C-labeled naphthalene (Figure S3, **1**), phenanthrene (**5**), and pyrene (**8**) were spiked into the first wash bottle before sampling. Mean recovery rates for these PAHs were 65 ± 13, 85 ± 18, and 80 ± 22%. Figure S2 also includes photos of five filters packed with glass wool after exhaust sampling at different thrust levels (1 h each). Samples at 85, 65, 30 and 7% thrust are compared with idle. While black soot particles were found at high thrust (85, 65%), brownish and oily particles dominated at low thrust (7%) and idle. Glass compartments and condensates were extracted with dichloromethane (DCM). Extracts were combined and concentrated. Aliquots of the extracts were mixed with deuterated PAH standards and fractionated by liquid chromatography (SiO_2_, *n*-hexane, DCM). Blank samples (*n* = 4) were also collected to determine background concentrations. PAHs were separated by gas chromatography (GC Mega 2, Rodano, Italy) on a capillary column (Restek, Bellefonte, USA, 30 m × 0.25 μm × 0.10 μm) and analyzed by mass spectrometry (MAT-95, Thermo Finnigan, Bremen, Germany). More detailed descriptions of the sample cleanup and analysis can be found elsewhere.^28,42^

### Assessment of the Genotoxic Potential

Of the many PAHs found in combustion-engine exhausts, some are well characterized with respect to their genotoxic potential. Figure S3 represents the chemical structures of 16 priority PAHs; eight of them, marked with asterisks, are carcinogens. Respective toxicity equivalence factors (TEFs) are indicated.^43,44^ Among the carcinogenic PAHs with a comparable mode-of-action, benzo(a)pyrene (**13**) is the most potent carcinogen, used as reference. A relative TEF of 1.0 is assigned to benzo(a)pyrene. Dibenz(ah)anthracene (**14**) is as carcinogenic (1.0×), while TEFs of other carcinogenic PAHs are one (0.1×), two (0.01×), or three orders (0.001×) of magnitude lower. The overall genotoxic potential (ng-TEQ/kg fuel) of an exhaust is calculated as the sum of the amounts of the eight carcinogenic PAHs multiplied by the respective TEFs.^43−45^ Background levels were calculated similarly using PAH background concentrations.

## Results and Discussion

### Fuel- and Thrust-Dependent Emissions of an Aircraft Jet Engine

**Figure 2:**
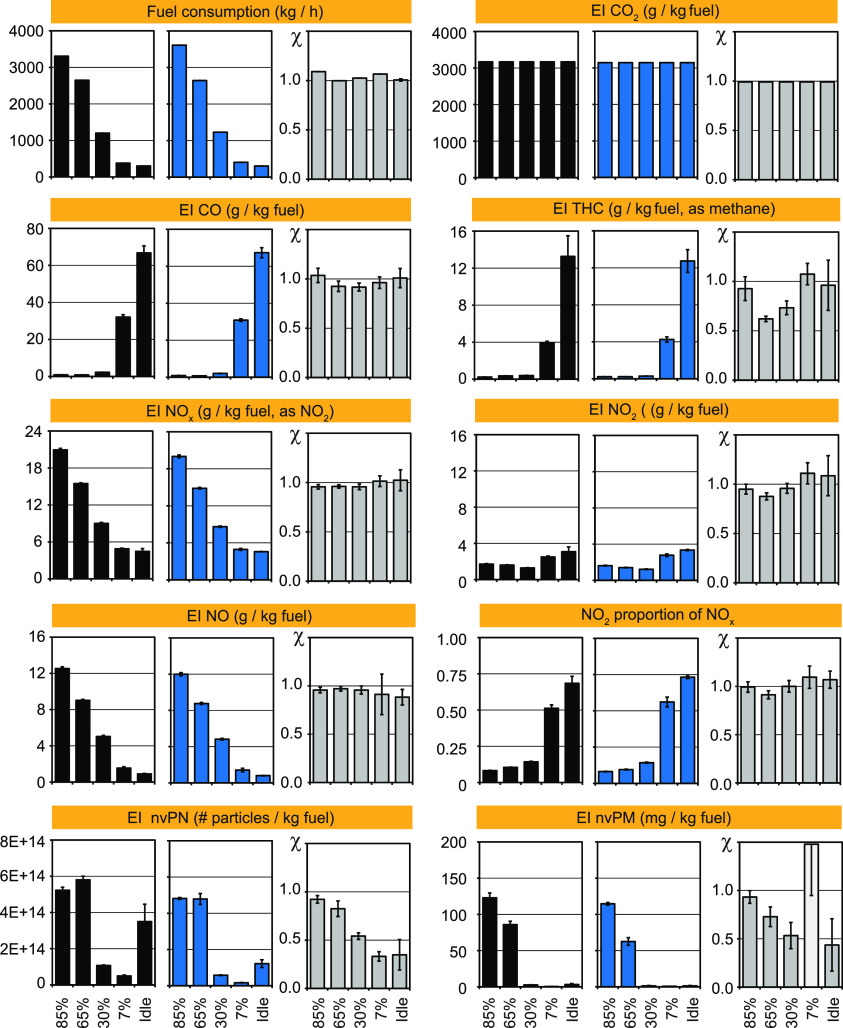
Fuel consumption (kg/h) and emission indices (EIs, g/kg fuel) of major exhaust constituents at different thrust levels. A widely used turbofan engine (CFM56–7B26) was operated at the SR-Technics facility at 85, 65, 30, 7% thrust and idle for 1 h per thrust level with Jet A-1 fuel (black) and a blend (blue) of hydro-processed fatty acids and esters (HEFA, 32%) and Jet A-1 fuel (68%). EIs of CO_2_, CO, THC (as CH_4_-equivalents), NO_*x*_ (as NO_2_-equivalents), NO, NO_2_, nvPM (mg/kg fuel), nvPN (#/kg fuel), and NO_2_proportions in NO_*x*_ (−) are shown. Fuel effects (χ, gray) for different compounds and thrust levels are displayed as dimensionless ratios of EI_HEFA-blend_/EI_Jet A-1 fuel_.

Emission indices (EIs g/kg of fuel) of major exhaust constituents at different thrust levels of a turbofan engine (CFM56–7B26) are compared in Figure 2. This engine type is one of the most widely used in the aviation industry (e.g., in Boeing 737–800 and 737-NextGen aircrafts) and can therefore be considered representative for current fleets. However, more recent aircrafts such as the Boeing-737-Max or Airbus 320-neo use newer engines with reduced fuel consumption and NOx emissions. The engine was first operated with Jet A-1 fuel (Figure 2, black) and later operated with a blend of hydro-processed fatty acids and esters (HEFA, 32%v, blue) and Jet A-1 fuel (68%v). Figure S1 displays the engine thrust-time diagram, exhaust gas temperatures, which varied from 370 to 560 °C and the fuel consumption per thrust level. Fuel consumption (kg/h) of the engine is enormous at 85% thrust, reaching 3309 and 3611 kg/h for Jet A-1 fuel and the HEFA blend, respectively. Fuel consumption decreased to 2647–2642, 1200–1231, 380–405, and 300–302 kg/h at 65, 30, 7% thrust, and idle.

EIs for CO_2_ of 3170 and 3142 g/kg of fuel were calculated for the Jet A-1 fuel and the HEFA blend. This results in the release of 10.5, 8.4, 3.8, 1.2, and 1.0 t CO_2_ per hour when operating the engine at 85, 65, 30, 7% thrust and idle. Based on the measured CO_2_ concentrations in the exhaust and the fuel H/C ratio, specific EIs for other compounds were calculated. Tables S3 and S4 report the respective data for the engine operated with Jet A-1 fuel and the HEFA blend. EIs for carbon monoxide (CO) were lowest at high thrust (85%) and highest at low thrust (7%) and idle, independent of the fuel (Figure 2). EIs of total hydrocarbons (THC, as methane equivalents) were also low at high thrust and high at idle. These findings indicate that combustion in the turbofan engine at low thrust is less efficient with increased CO and THC emissions. This is a common attribute of the rich-burn, quick-mix, and lean-burn (RQL) combustion employed in this engine.

This is further confirmed when NO_*x*_ and NO emissions are considered (Figure 2). Highest NO_*x*_ emissions (as NO_2_ equivalents) of 20.9 and 20.0 g/kg of fuel were found for the engine at 85% thrust with Jet A-1 fuel and the HEFA blend. NO_*x*_ emissions decreased to 15.5–14.9, 9.0–8.6, 4.9–4.9, and 4.5–4.6 g/kg fuel at 65, 30, 7% thrust, and idle, respectively. Most of the NO_*x*_ released at high thrust is nitric oxide (NO). NO emissions decreased by 1 order of magnitude from 12.5 to 12.0 at high thrust (85%) to 0.9–0.8 g/kg fuel at idle. Interestingly, direct NO_2_ emissions of the jet engine varied only little, from 1.2 to 3.4 g/kg from 85% thrust to idle, independent of the fuel (Tables S3, S4). In other words, lowest NO_2_-proportions of 0.08–0.10 were found at 85 and 65% thrust and highest NO_2_-proportions of 0.51–0.56 and 0.68–0.73 were obtained at 7% thrust and idle. Thus, NO_2_proportions at ground operation are 5–8× higher than those at cruise.

Highest particle mass emissions (nvPM) of 123 and 86 mg/kg of fuel were found at 85 and 65% thrust. These particles were black as shown in Figure S2, while particles at idle and low thrust were brownish and oily. Respective emissions were minimal at 0.5–3.3 mg/kg fuel. Particle number (nvPN) emissions followed the same trend. EIs of 5.2 and 5.8 × 10^14^ particles/kg fuel were found at 85 and 65% thrust (Figure 2). At lower thrust of 30 and 7%, EIs dropped to 1.1 and 0.5 × 10^14^ particles/kg of fuel and increased to 3.5 × 10^14^ particles/kg at idle. Particle data presented here were not corrected for losses in sampling lines. Dependent on particle size, such losses can be relevant as can be seen from corrected data (Tables S4 and S5).^39^ Correction factors varied from 1.2 to 2.2 and 2.7–7.3 for nvPM and nvPN emissions, respectively.

Considering the photographs (Figure S2) of low- and high-thrust particles and the contradicting trends for CO, THC, and NO, we conclude that particles released at different thrust levels are remarkably different. High-thrust particles are abundant, small (20–30 nm), black, and released together with NO, while low-thrust particles are less abundant, smaller (10–20 nm), brownish, and released together with CO and hydrocarbons. Upon cooling, these particles adsorb semivolatile hydrocarbons to produce the oily look (Figure S2).

Emission trends for the HEFA blend (Figure 2, blue) and Jet A-1 fuel (black) are similar. Figure 2 (gray) also displays fuel effects (χ), which are deduced from the EI ratios of the engine operated with the HEFA blend or Jet A-1 fuel. Ratios <1 indicate that emissions with the HEFA blend are lower than with Jet A-1 fuel. Fuel effects were small for pollutants like NO, NO_2_, and CO. Nevertheless, they are large for nvPN, with χ = 0.92, 0.82, 0.54, 0.33, and 0.35 at 85, 65, 30, 7% thrust and idle, respectively. As strong fuel-effects of χ = 0.93, 0.73, 0.53, and 0.44 were observed for nvPM at 85, 65, 30% thrust and idle, respectively, with one outlier (χ = 1.48) at 7% thrust, where PM emissions were minimal for both fuels and uncertainties large (Figure 2). As discussed before, differences of smoke point measurements for Jet A-1 fuel of 21.9 ± 0.5 mm and for the HEFA blend of 23.0 ± 0.6 mm are relatively small. One can conclude that blending Jet A-1 fuel with HEFA has much stronger effects on nvPN and the chemical nature of low-thrust particles, which appear brownish and not black as high-thrust particles than on smoke point (Figure S2).

### Thrust-Dependent Emissions of Polycyclic Aromatic Hydrocarbons

**Figure 3:**
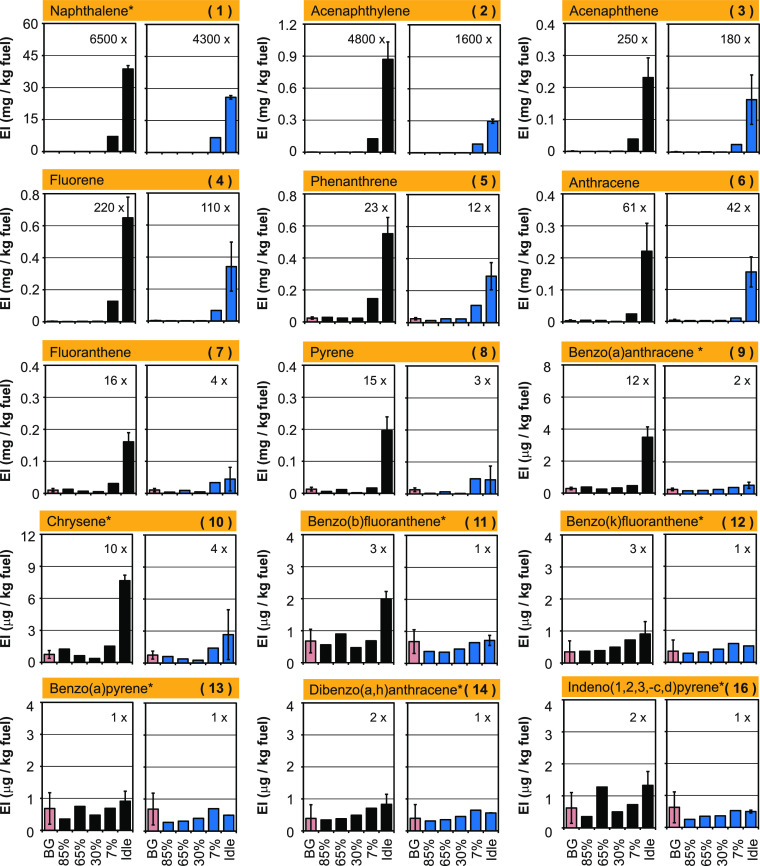
Emission indices (EI, mg, or μg/kg fuel) of priority PAHs at different thrust levels. A turbofan engine (CFM56–7B26) was operated with Jet A-1 fuel (black) and a blend (blue) of hydro-processed fatty acids and esters (HEFA, 32%) and Jet A-1 fuel (68%) at 85, 65, 30, 7% thrust and idle for 1 h per thrust level. EIs and chemical structures of PAHs are also provided in the Supporting Information. Genotoxic PAHs are marked with asterisks. Mean EIs at idle for Jet A-1 fuel (black, *n* = 3) and the HEFA blend (blue, *n* = 2) are compared with mean background values (BG, pink, *n* = 4) and respective ratios are indicated.

Chemical structures of the 16 priority PAHs and their numbering are given in Figure S3. Figure 3 and Tables S6 and S7 (supporting material) display EIs of priority PAHs including eight genotoxic PAHs. EIs of abundant 2- to 4-ring PAHs (**1**–**8**) are reported in mg/kg fuel, and those of genotoxic 4–6-ring PAHs (**9**–**16**) are reported in μg/kg fuel. In other words, the 2-ring PAH naphthalene (**1**) is 4 orders of magnitude more abundant in jet engine exhaust at idle than the 5-ring (**13**, **14**) and 6-ring PAHs (**15**, **16**).

Highest naphthalene (**1**) emissions of 39 and 7.1 mg/kg of fuel were found at idle and 7% thrust, when operating the engine with Jet A-1 fuel (Figure 3, black). Naphthalene emissions at 85, 65, and 30% thrust were 0.01, 0.05, and 0.04 mg/kg, respectively, thus 3 orders of magnitude lower than at idle. Emissions of the three-ring PAHs acenaphthylene (**2**), acenaphthene (**3**), fluorene (**4**), phenanthrene (**5**), and anthracene (**6**) were maximal at idle with 0.87, 0.23, 0.65, 0.55, and 0.02 mg/kg fuel. At 7% thrust, EIs of these 3-ring PAHs reached about 11–26% of the idle level. At higher thrust, 3-ring PAH emissions further decreased to <5% of the idle level. EIs of the carcinogenic 4-ring PAHs benzo(a)anthracene (**9**) and chrysene (**10**) of 3.5 and 7.6 μg/kg fuel are maximal at idle too. High-thrust emissions of 4-ring PAHs are close to background levels (Figure 3, pink). Differences to background levels become even smaller for five- and six-ring PAHs (**11**–**16**). Respective EIs at idle varied from 1 to 3 μg/kg fuel, which is at or slightly above background levels. PAHs have become ubiquitous air pollutants, released by various combustion-related processes. Therefore, we have also evaluated background levels of our sampling devices (*n* = 4) and compared them with levels of exhaust samples. Respective ratios are listed in Figure 3.

To summarize, EIs of 2- to 4-ring PAHs (**1**–**10**) at idle are 1–3 orders of magnitude (10×–6500×) above background levels (Figure 3). Thus, most of the 2- to 4-ring PAHs (**1**–**10**) are released during ground operation of the jet engine. The emission characteristics of these PAHs and the ones of other HCs (Figure 2) is similar. Emissions of 2- to 4-ring PAHs (**1**–**10**) are minimal at high thrust, as expected for a higher combustion efficiency in the high-NO_*x*_ regime.

### Influence of Biofuel on PAH Emissions and the Genotoxic Potential of Jet Engine Exhaust

Combustion of a blend of HEFA (32%v) and Jet A-1 fuel (68%v) had only small effects (χ) on fuel consumption, CO_2_, CO, THC, and NO emissions, which varied from χ = 0.8–1.2 (Figure 2, gray). Nevertheless, if such fuels originate from fatty acids and esters of oil plants such as sunflower, rapeseed, and others or waste fat and oil of the food industry, such fuels can be considered as renewable biofuels, lowering overall CO_2_ emissions of a jet engine.

**Figure 4:**
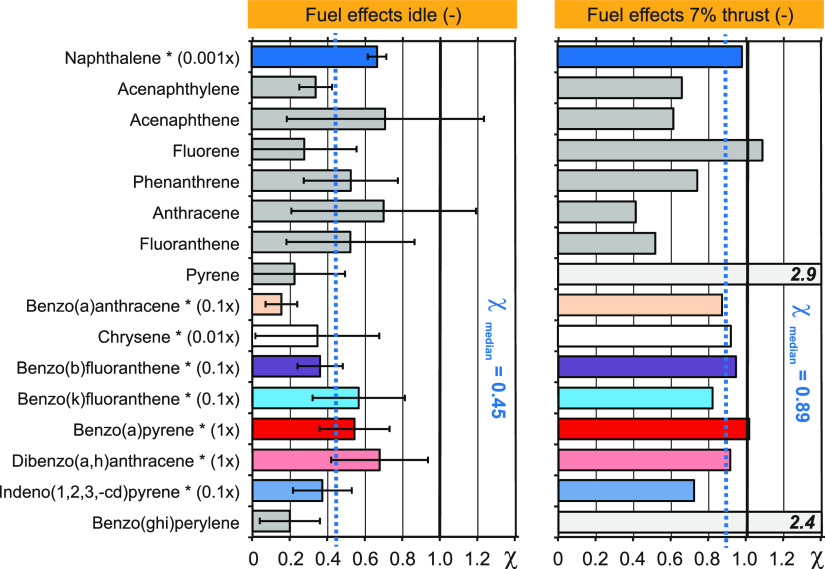
Fuel effects (χ) of priority PAHs of a turbofan engine (CFM56–7B26) operated at idle (left) and 7% thrust (right). Fuel effects of individual PAHs are reported as dimensionless ratios of emission indices EI_HEFA blend_/EI_Jet A-1 fuel_. Values <1 indicate a reduction of specific PAH emissions. Median values (dashed blue lines) and genotoxic PAHs (asterisk) are displayed in color. Respective toxicity equivalence factors are indicated.

Significant fuel effects were mostly observed at idle and low thrust, not at high thrust, for nvPN, nvPM (Figure 2, gray), and for all priority PAHs (Figure 3, blue). Figure 4 displays fuel effects for priority PAHs at idle and 7% thrust, where PAH emissions are highest. Table S8 lists the respective data. At idle, χ-values were always <1, indicating a net reduction of PAH emissions with HEFA. A median fuel effect χ = 0.45 was determined for the 16 priority PAHs at idle (Figure 4). At 7% thrust, median fuel effects were smaller at χ = 0.89 with two outliers. This compares with nvPN fuel effects of χ = 0.35 and 0.33 at idle and 7% thrust. Thus, blending Jet A-1 fuel with HEFA and with it increasing the paraffin and lowering the aromatics content induced a reduction of PAH and particle emissions.

### Genotoxic Potential of Jet Engine Exhausts

**Figure 5:**
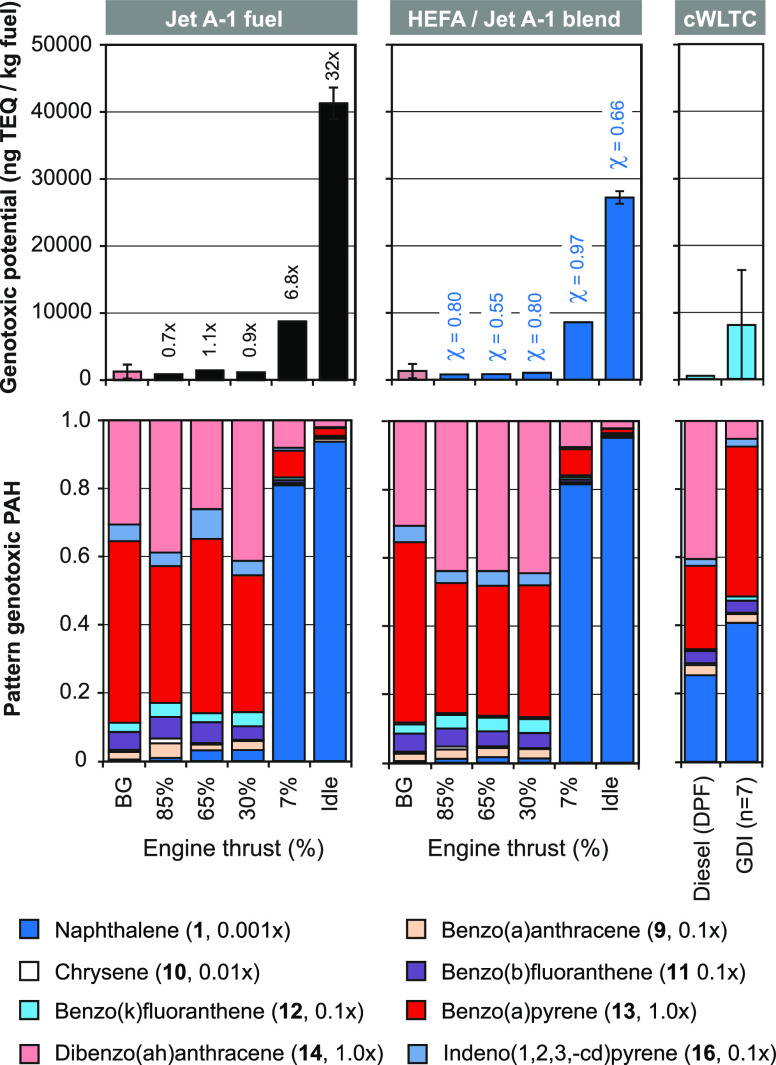
Genotoxic potential (ng-TEQ/kg fuel) and pattern of genotoxic PAHs in jet engine exhausts at different thrust levels with Jet A-1 fuel (black) and the HEFA blend (blue). For comparison, the genotoxic potentials of exhausts from gasoline-direct-injection (GDI) vehicles (*n* = 7, Euro-3 to -6) and a diesel vehicle with particle filter (Euro-5, Peugeot 4008) in the cold started WLTC are given (light blue). Color codes and the toxicity-equivalence factors (TEFs) of genotoxic PAHs are indicated. The numbers indicated are multiples of background levels (black) and fuel effects (χ, blue) induced by HEFA-blending.

Figure 5 displays the genotoxic potential (ng-TEQ/kg fuel) of jet engine exhausts at different thrust levels when operated with Jet A-1 fuel (black) and the HEFA blend (blue). It is assumed that the eight genotoxic PAHs (Figure S3) induce cancer in humans in a comparable mode of action. A set of toxicity-equivalence factors (TEFs) has been established for these genotoxic PAHs and used here.^43−45^ TEFs of genotoxic PAHs in relation to benzo(a)pyrene (**13**) with a TEF of 1.0 are indicated, and sums of TEF-weighted EIs are compared. The highest genotoxic potential of 41300 ng-TEQ/kg fuel is found at idle, when Jet A-1 fuel is used (Figure 5, black). Blending with the HEFA fuel lowered the genotoxic potential at idle by 34% to 27200 ng-TEQ/kg fuel. Genotoxic potentials further decreased to 8800 and 8500 ng-TEQ/kg of fuel at 7% thrust with Jet A-1 fuel and the HEFA blend. Respective fuel effects are χ = 0.66 and 0.97. Genotoxic potentials at high thrust varied from 900 to 1400 ng-TEQ/kg fuel. However, these values are close to the background level of 1300 ng-TEQ/kg fuel.

For comparison, the mean (*n* = 7) genotoxic potential of a fleet of gasoline-direct injection (GDI) vehicles (Euro-3 to -6) at transient vehicle operation in the worldwide harmonized light-duty vehicle test cycle (WLTC) of 8000 ng-TEQ/kg fuel is also given (Figure 5).^27^ Mean PN emissions of the GDI-vehicles were 4.2 × 10^13^ particles/kg fuel (23 nm cutoff). This compares to PN emissions of the jet engine of 0.5–5.2 × 10^14^ particles/kg of jet fuel (Figure 2). The genotoxic potential of a diesel vehicle (Euro-5, Peugeot 4008) with a particle filter (DPF) is 500 ng-TEQ/kg fuel (Figure 5). Respective PN emissions were 8.2 × 10^11^ particles/kg fuel.^27^ In other words, the genotoxic potential of jet engine exhaust with Jet A-1 fuel at idle is 5- and 90-times higher than the ones of the GDI fleet and the diesel vehicle with a particle filter. In addition, PN emissions of the jet engine at idle were 8 and 400 times higher than those of the GDI and diesel vehicles.^27^

Figure 5 also displays the TEF-weighted pattern of genotoxic PAHs of jet engine exhausts at different thrust levels and fuels. The patterns at idle and 7% thrust are similar and dominated by naphthalene (blue), which accounts for 94–95 and 81–82% of the TEQ for both fuels. Other PAHs contributed only 5–19% to the overall genotoxic potential at ground operation. At 65 and 85% thrust, mainly benzo(a)pyrene (**13**, red) and dibenzo(ah)anthracene (**14**, pink) contribute 79–84% to the genotoxic potential. Patterns with the HEFA blend are similar at all thrust levels. Figure 5 also displays genotoxic PAH pattern found in GDI (*n* = 7) and diesel vehicle exhausts.^27^ Both patterns include relevant contributions of naphthalene (**1**, blue) of 41 and 25% and benzo(a)pyrene (**13**, red) of 44 and 25%. With this, they fall between high- and low-thrust patterns of the jet engine.

### Environmental and Health Impact of Coreleased Jet Engine Particles and Genotoxic PAHs

Civil aviation experienced substantial annual growth rates before the COVID pandemic that was associated with increasing CO_2_ emissions. In this respect, renewable jet fuels such as HEFA, replacing fossil-based fuels, are interesting alternatives to lower the CO_2_ emissions of the aviation industry.

The release of large numbers of ultrafine particles is another consequence of jet engine applications. These small nanoparticles are injected in all layers of the troposphere and the lower stratosphere with still unclear impacts on health, climate, and the environment. Jet engine particles are small, with diameters of 10–30 nm.^37,39^ They are even smaller than diesel and GDI particles with diameters of 70–100 nm.^9,27^ PN emissions of this jet engine varied from 0.5 to 5.8 × 10^14^ particles/kg fuel (10 nm cutoff), those of GDI and diesel vehicles were 1–3 orders of magnitude lower at 4.2 × 10^13^ and 8.2 × 10^11^ particles/kg fuel, respectively (23 nm cutoff).^25^ Jet engine particles released at low thrust and idle have different light absorption and scattering properties than high-thrust particles.^36^ High-thrust particles are black with little adsorbates, while idle particles appear brownish and oily with more adsorbates (Figure S2). It is expected that surface-rich jet engine particles coated with non- and semivolatile compounds like PAHs also affect atmospheric chemistry downwind of airports.

The genotoxic potential of jet engine exhausts was highest at idle and 7% thrust. Thus, the many jet engine particles released at ground operation carry 30 and 7 times more genotoxic material than particles released during climb-out and cruising. This is relevant from an occupational health point of view. The increased genotoxic potential of jet engine particles at ground operation leads to higher exposures of ground personnel, passengers, and residents living nearby airports.

Blending fossil jet fuel with the HEFA biofuel reduced the emissions of particles and their genotoxic potential. Fuel effects were strongest at the ground operation. Therefore, it is efficient to use costly biofuels in idle and taxi operations, where fuel consumption is low and fuel effects are strongest. However, this dual fuel approach with high quality fuels for ground operation and standard fuels for high thrust operations would require considerable investments in the infrastructure of airports and airplanes. However, e.g., for short distance flights of less than 1 h, such high quality fuels can be a reasonable option.

Better fuels are also important measures to abate the Trojan horse effect, which describes the observation that persistent soot nanoparticles transport genotoxic adsorbates into the human lung and even beyond the alveolar membrane to every organ of the body.^15−18^

The use of high-quality fuels with, e.g., low sulfur and ash contents, has become mandatory in certain marine ports, shipways, and dedicated low emission zones to minimize exposure of port workers or residents. The use of paraffin-rich fuels for hand-held two-stroke engines such as chain saws was a major step, lowering emissions of genotoxic compounds and particles.^46^ These applications show the potential of alternative fuels with high proportions of paraffins and reduced levels of aromatics. If paraffin-rich jet fuels can be produced at large quantities in renewable ways, emissions of particles, genotoxic PAHs, and CO_2_ of the aviation industry can be lowered in the future. We conclude that using such paraffin-rich fuels at airports is a promising strategy to improve air quality and reduce the exposure of personnel, passengers, and residents living near airports.

## Supporting Information Available

Figures S1 and S2: information on engine operation and sampling; Figure S3: chemical structures of PAHs; Tables S1 and S2: information on both fuels; and Tables S3–S8: quantitative results of regulated pollutants, priority PAHs, and fuel effects(PDF)
