# Supplementary material for: Corelease of Genotoxic Polycyclic Aromatic Hydrocarbons and Nanoparticles from a Commercial Aircraft Jet Engine – Dependence on Fuel and Thrust
Source: Environ Sci Technol. 2024 Jan 11;58(3):1615–24. doi: 10.1021/acs.est.3c08152 (PMC10809754; doi:10.1021/acs.est.3c08152)
Supplement: Supplementary file 1 — es3c08152_si_001.pdf [file es3c08152_si_001.pdf]

Co-Release of Genotoxic PAHs and Nanoparticles from a Commercial Aircraft Jet Engine – Dependence on Fuel and Thrust

Norbert V. Heeb[a], Maria Muñoz[a,1], Regula Haag[a], Simon Wyss[b,2], David Schönenberger[a,b], Lukas Durdina[a,3] , Miriam Elser[a,c], Frithjof Siegerist[d], Joachim Mohn[b], Benjamin T. Brem[a,4].

[a]Empa, Swiss Federal Laboratories for Materials Science and Technology, Laboratory for Advanced Analytical Technologies, Überlandstrasse 129, CH-8600 Dübendorf, Switzerland

[b]Empa, Swiss Federal Laboratories for Materials Science and Technology, Laboratory for Air Pollution/Environmental Technology, Überlandstrasse 129, CH-8600 Dübendorf, Switzerland

[c]Empa, Swiss Federal Laboratories for Materials Science and Technology, Automotive Powertrain Technologies Laboratory, Überlandstrasse 129, CH-8600 Dübendorf, Switzerland

[d]SR Technics Switzerland AG, Zurich-Airport, CH-8058 Kloten, Switzerland

Present address:

[1]M.M.: Arcadis, Ifangstrasse, CH-8952 Schlieren, Switzerland

[2]S.W.: FIZ, Forensic Institute Zurich, Police and Justice Center, Güterstrasse 33, CH-8010 Zürich, Switzerland

[3]L.D.: ZHAW Zurich, University of Applied Sciences, Centre for Aviation, Technikumstrasse 71, CH-8401 Winterthur, Switzerland

[4]B.T.B.: Paul Scherrer Institute, Laboratory of Atmospheric Chemistry, CH-5232 Villigen, Switzerland

Corresponding Author
Norbert Heeb. Empa, Swiss Federal Laboratories for Materials Science and Technology, Laboratory for Advanced Analytical Technologies, Überlandstrasse 129, CH-8600 Dübendorf, Switzerland. Phone: +41 58 765 4257. e-mail: norbert.heeb@empa.ch.
orcid.org/0000-0002-6133-4421

Supporting Information Available

Additional figures and tables can be found in Supporting Information. **Figures S1** and **S2** give information on engine operation and sampling. **Figure S3** displays chemical structures of PAHs. **Tables S1** and S2 provide information on both fuels and **Tables S3-S8** report quantitative results of regulated pollutants, priority PAHs and fuel effects. This material is available free of charge via the Internet at http://pubs.acs.org.

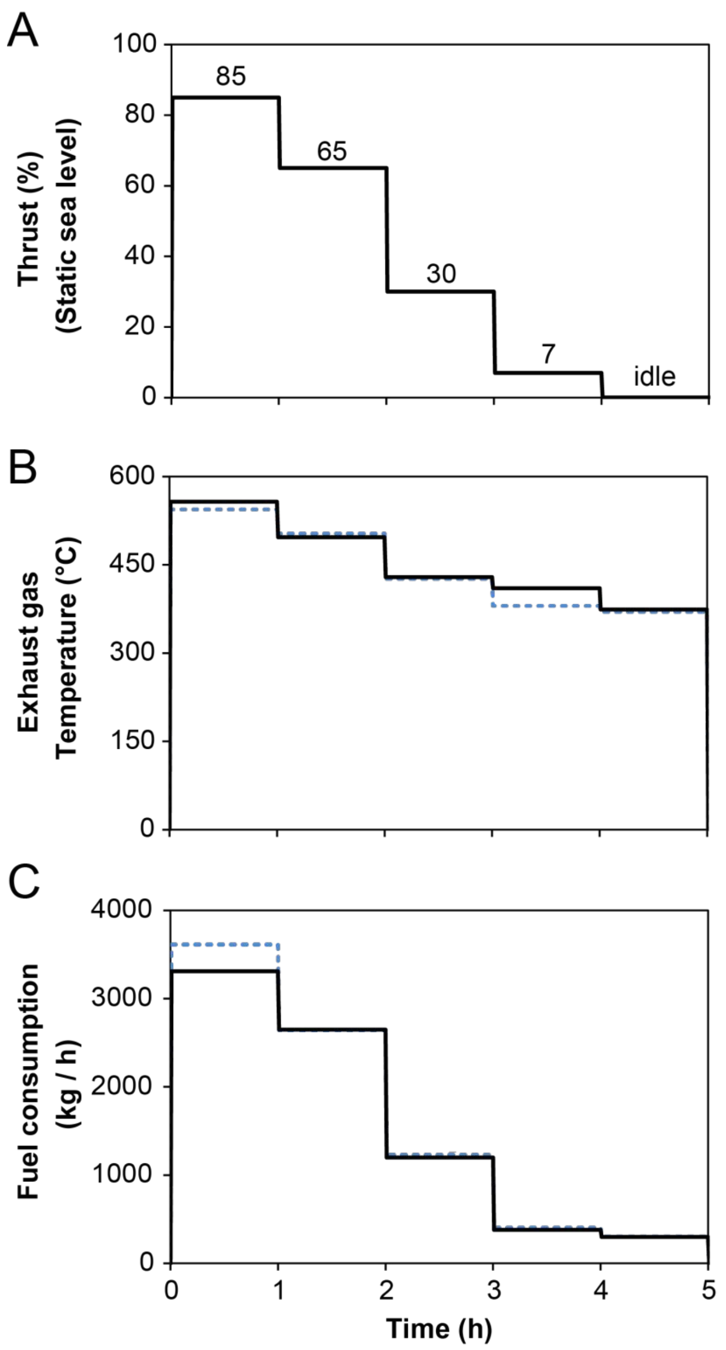

Figure S1. Test cycle, average exhaust gas temperatures and fuel consumption. The jet engine (CFM56-7B26) was operated at different thrust levels (A, %) of 85, 65, 30 and 7% thrust and idle. After equilibration, five exhaust samples were collected with the GenToxJet sampling system for one hour at each stage. The engine was first operated with Jet A-1 fuel, followed by the HEFA blend. Exhaust gas temperatures (B, ˚C) and fuel consumption (C, kg/h) during different cycle phases using Jet A-1 fuel (black solid) and the HEFA blend (blue dashed) are also given.

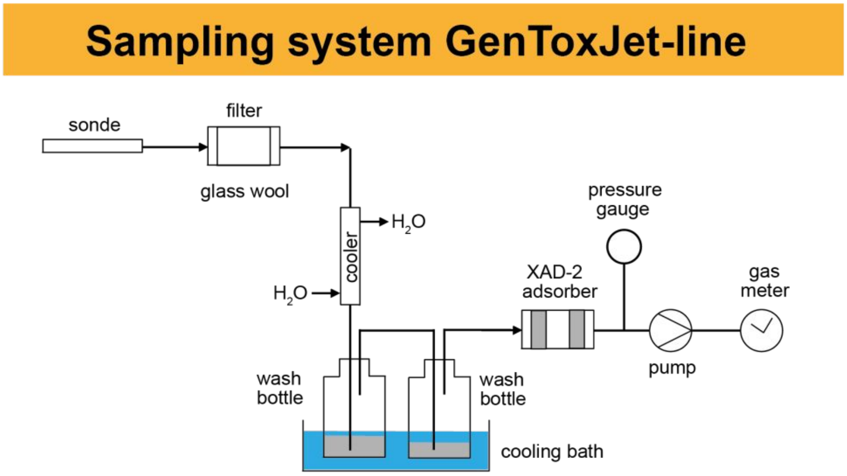

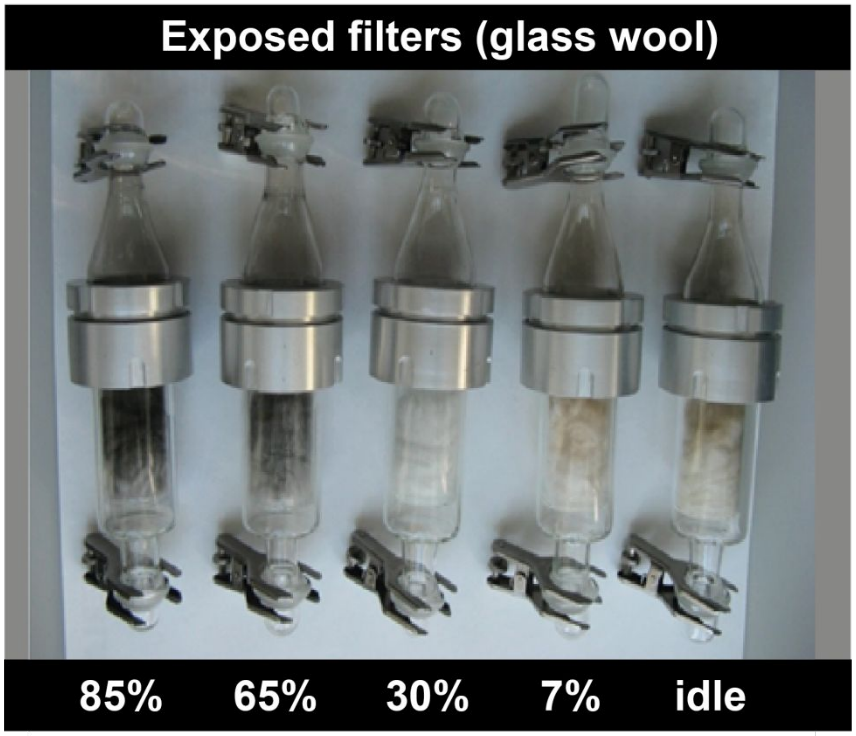

Figure S2. All-glass sampling devises used at the GenToxJet-line. The devices include a filter (glass wool), cooler, two wash bottles kept at ~0˚C, two-stage XAD adsorber, pump and a gas meter. In addition, photos of five filters packed with glass wool are shown after sampling at different thrust levels (1h each). Samples at 85, 65, 30 and 7% thrust are compared with idle. While black soot particles were found at high thrust (85, 65%), brownish and oily particles dominated at low thrust (7%) and idle.

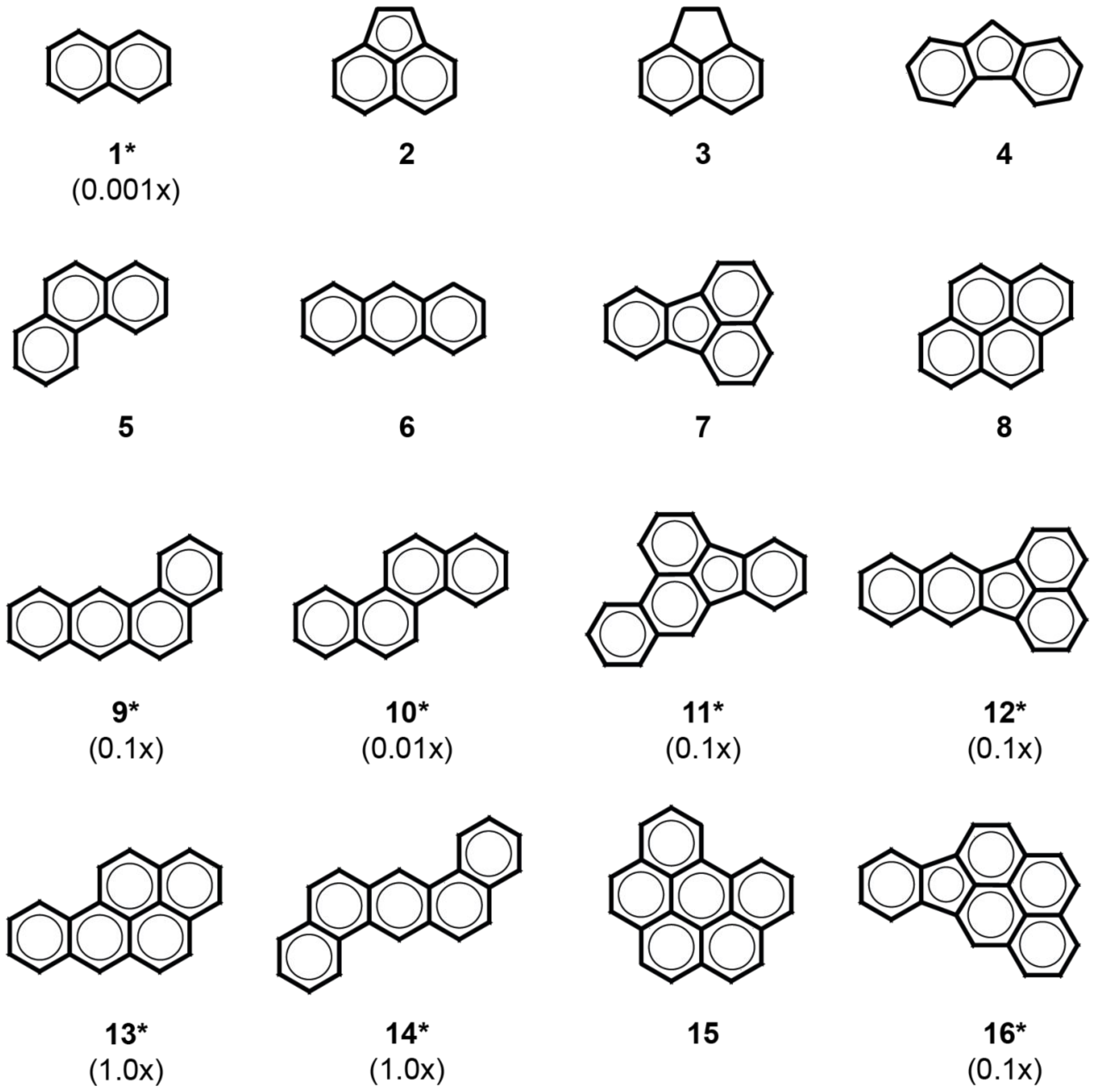

Figure S3. Chemical structures of priority PAHs. The following PAHs were studied: naphthalene (**1**, 0.001x), acenaphtylene (**2**), acenaphtene (**3**), fluorene (**4**), phenanthrene (**5**), anthracene (**6**), fluoranthene (**7**), pyrene (**8**), benzo(a)anthracene (**9,** 0.1x), chrysene (**10**, 0.01x), benzo(b)fluoranthene (**11**, 0.1x), benzo(k)fluoranthene (**12**, 0.1x), benzo(a)pyrene (**13**, 1.0x), dibenz(ah)anthracene (**14**, 1.0x), benzo(ghi)perylene (**15**), indeno(1,2,3-cd) pyrene (**16**, 0.1x). Eight PAHs are carcinogens to humans (asterics). Respective toxicity-equivalence factors (TEFs) in relation to benzo(a)pyrene (**13**, 1.0x) are given in brackets.

Table S1. Fuel Characteristics of Jet A-1 Fuel (Mean, n=2)
(Specification Joint Fuelling System Check List Jet A-1 (AFQRJOS)

Property	Unit	Fuel	(std)	Limit (low)	Limit (high)	Test Method
Aromatics	% (V/V)	17.8	0.3		25.0	ASTM D 1319
Sulfur content, total	% (m/m)	0.059	0.005		0.30	ASTM D 5453
Distillation (101.3 kPa)						ASTM D 86
Initial boiling point	°C	150.5	0.5			
10 Vol% recovered at	°C	165.0	1.0		205.0	
20 Vol% recovered at	°C	171.5	0.5			
50 Vol% recovered at	°C	190.5	0.5			
90 Vol% recovered at	°C	235.0	1.0			
End point	°C	258.5	0.5		300.0	
Residue	% (V/V)	1.1	0.0		1.5	
Loss	% (V/V)	0.7	0.1		1.5	
Viscosity at -20 °C	mm2/s	3.430	0.021		8.000	ASTM D 445
Specific energy, net	MJ/kg	43.30	0.00	42.80		ASTM D 3338
Smoke point	mm	21.9	0.5	18.0		ASTM D 1322
Naphthalenes	% (V/V)	0.84	0.03		3.00	ASTM D 1840
Densitiy at 15 °C	kg/m3	795.1	0.7	775.0	840.0	ASTM D 4052
Hydrogen	% (m/m)	13.8	0.1			ASTM D 5291

Table S2. Fuel Characteristics of HEFA (32%) and Jet A-1 Fuel (68%) Blend (Mean, n=2)
(Specification Joint Fuelling System Check List Jet A-1 (AFQRJOS)

Property	Unit	Fuel	(std)	Limit (low)	Limit (high)	Test Method
Aromatics	% (V/V)	11.3	0.1		25.0	ASTM D 1319
Sulfur content, total	% (m/m)	0.035	0.000		0.30	ASTM D 5453
Distillation (101.3 kPa)						ASTM D 86
Initial boiling point	°C	154.5	1.5			
10 Vol% recovered at	°C	168.5	0.5		205.0	
20 Vol% recovered at	°C	174.0	0.0			
50 Vol% recovered at	°C	194.5	0.5			
90 Vol% recovered at	°C	237.0	0.0			
End point	°C	255.5	0.5		300.0	
Residue	% (V/V)	1.2	0.0		1.5	
Loss	% (V/V)	0.5	0.1		1.5	
Viscosity at -20 °C	mm^2/s	3.677	0.002		8.000	ASTM D 445
Specific energy, net	MJ/kg	43.60	0.00	42.80		ASTM D 3338
Smoke point	mm	23.0	0.6	18.0		ASTM D 1322
Naphthalenes	% (V/V)	0.54	0.02		3.00	ASTM D 1840
Densitiy at 15 °C	kg/m3	781.8	0.0	775.0	840.0	ASTM D 4052
Hydrogen	% (m/m)	14.3	0.1			ASTM D 5291

Table S3. Emission Indices (EI) of Regulated Pollutants with Jet A-1 Fuel

	Thrust [a)]	T-85%		T-65%		T-30%		T-7%		Idle	
		mean	std	mean	std	mean	std	mean	std	mean	std
Fuel consumption	kg/h	**3309**		**2647**		**1200**		**380**		**300**	3
EI CO_2	g/kg fuel	**3170**		**3170**		**3170**		**3170**		**3170**	
EI CO	g/kg fuel	**0.90**	0.03	**0.83**	0.02	**2.27**	0.05	**32.10**	1.26	**66.76**	3.78
EI THC	g/kg fuel, as methane	**0.24**	0.01	**0.36**	0.00	**0.40**	0.02	**3.96**	0.14	**13.26**	2.23
EI NO_x	g/kg fuel, as NO_2	**20.93**	0.25	**15.46**	0.13	**9.01**	0.15	**4.88**	0.11	**4.47**	0.44
EI NO_2	g/kg fuel	**1.71**	0.05	**1.61**	0.03	**1.28**	0.03	**2.50**	0.11	**3.08**	0.52
EI NO	g/kg fuel	**12.53**	0.20	**9.03**	0.10	**5.04**	0.12	**1.55**	0.14	**0.91**	0.05
NO_2 proportion of NO_x	(-)	**0.082**	0.003	**0.104**	0.002	**0.142**	0.005	**0.51**	0.02	**0.68**	0.05
EI nvPM [b)]	mg/kg fuel	**122.9**	6.7	**86.0**	4.6	**2.8**	0.1	**0.5**	0.1	**3.3**	1.3
EI nvPM [c)]	mg/kg fuel	**196.6**	42.6	**102.7**	22.2	**4.2**	0.9	**1.2**	0.3	**5.1**	1.1
Particle loss correction factor	(-)	**1.6**		**1.2**		**1.5**		**2.2**		**1.5**	
EI nvPN [b,d)]	# particles/kg fuel	**5.2E+14**	1.6E+13	**5.8E+14**	2.0E+13	**1.1E+14**	2.7E+12	**5.0E+13**	4.6E+12	**3.5E+14**	9.6E+13
EI nvPN [c,d)]	# particles/kg fuel	**1.5E+15**	3.9E+14	**1.6E+15**	4.2E+14	**4.6E+14**	1.2E+14	**3.0E+14**	7.9E+13	**2.0E+15**	5.4E+14
Particle loss correction factor	(-)	**2.9**		**2.7**		**4.3**		**6.1**		**5.9**	

a) Sea level thrust
b) Without particle loss corrections
c) With particle loss corrections
d) Particle cut-off at 10 nm

Table S4. Emission Indices (EI) of Regulated Pollutants with HEFA (32%v) and Jet A-1 Fuel (68%v) Blend

	Thrust [a]	T-85%		T-65%		T-30%		T-7%		Idle	
		mean	std	mean	std	mean	std	mean	std	mean	std
Fuel consumption	kg/h	**3611**		**2642**		**1231**		**405**		**302**	1
EI CO_2	g/kg fuel	**3142**		**3142**		**3142**		**3142**		**3142**	
EI CO	g/kg fuel	**0.94**	0.03	**0.77**	0.02	**2.09**	0.05	**30.89**	0.67	**67.36**	2.71
EI THC	g/kg fuel, as methane	**0.22**	0.02	**0.22**	0.01	**0.29**	0.01	**4.25**	0.27	**12.74**	1.23
EI NO_x	g/kg fuel, as NO_2	**20.02**	0.23	**14.85**	0.13	**8.62**	0.11	**4.94**	0.15	**4.58**	0.03
EI NO_2	g/kg fuel	**1.63**	0.04	**1.41**	0.03	**1.23**	0.04	**2.77**	0.15	**3.35**	0.06
EI NO	g/kg fuel	**11.99**	0.17	**8.76**	0.11	**4.82**	0.09	**1.41**	0.20	**0.80**	0.02
NO_2 proportion of NO_x	(-)	**0.081**	0.002	**0.095**	0.002	**0.142**	0.004	**0.56**	0.03	**0.73**	0.01
EI nvPM [b]	mg/kg fuel	**114.7**	1.9	**62.6**	5.3	**1.5**	0.3	**0.8**	0.1	**1.4**	0.3
EI nvPM [c]	mg/kg fuel	**163.8**	35.5	**85.2**	18.5	**2.1**	0.5	**1.2**	0.2	**2.5**	0.6
Particle loss correction factor	(-)	**1.4**		**1.4**		**1.4**		**1.5**		**1.8**	
EI nvPN [b,d]	# particles/kg fuel	**4.8E+14**	5.4E+12	**4.8E+14**	3.0E+13	**5.8E+13**	2.2E+12	**1.6E+13**	8.9E+11	**1.2E+14**	2.2E+13
EI nvPN [c,d]	# particles/kg fuel	**1.3E+15**	3.5E+14	**1.4E+15**	3.7E+14	**3.1E+14**	8.2E+13	**1.2E+14**	3.2E+13	**7.7E+14**	2.0E+14
Particle loss correction factor	(-)	**2.8**		**2.9**		**5.4**		**7.3**		**6.3**	

a) Sea level thrust
b) Without particle loss corrections
c) With particle loss corrections
d) Particle cut-off at 10 nm

Table S5. Fuel Effects (χ) for Regulated Pollutants [a)]

	Thrust [b)]	T-85%		T-65%		T-30%		T-7%		Idle	
		mean	std	mean	std	mean	std	mean	std	mean	std
Fuel consumption	(-)	**1.09**		**1.00**		**1.03**		**1.07**		**1.01**	0.01
EI CO_2	(-)	**0.99**		**0.99**		**0.99**		**0.99**		**0.99**	
EI CO	(-)	**1.04**	0.07	**0.92**	0.05	**0.92**	0.04	**0.96**	0.06	**1.01**	0.10
EI THC	(-)	**0.93**	0.12	**0.62**	0.03	**0.73**	0.07	**1.07**	0.11	**0.96**	0.25
EI NO_x	(-)	**0.96**	0.02	**0.96**	0.02	**0.96**	0.03	**1.01**	0.05	**1.02**	0.11
EI NO_2	(-)	**0.95**	0.05	**0.88**	0.04	**0.96**	0.05	**1.11**	0.11	**1.09**	0.20
EI NO	(-)	**0.96**	0.03	**0.97**	0.02	**0.96**	0.04	**0.91**	0.21	**0.88**	0.08
NO_2 proportion of NO_x	(-)	**0.99**	0.05	**0.91**	0.04	**1.00**	0.06	**1.10**	0.12	**1.07**	0.09
EI nvPM [c)]	(-)	**0.93**	0.07	**0.73**	0.10	**0.53**	0.14	**1.48**	0.53	**0.44**	0.27
EI nvPM [d)]	(-)	**0.83**		**0.83**		**0.50**		**0.97**		**0.50**	0.27
EI nvPN [c,e)]	(-)	**0.92**	0.04	**0.82**	0.08	**0.54**	0.03	**0.33**	0.05	**0.35**	0.16
EI nvPN [d,e)]	(-)	**0.90**		**0.88**		**0.68**		**0.40**		**0.38**	

a) Dimensionless ratio of $EI_{HEFA\text{-}blend}$ / $EI_{Jet\ A\text{-}1\ fuel}$
b) Sea level thrust
c) Without particle loss corrections
d) With particle loss corrections
e) Particle cut-off at 10 nm

Table S6. Emission Indices (EI) for Priority PAHs with Jet A-1 Fuel [a)]

	TEF	Thrust [b)]	Background mean (n=4)	std	T-85%	T-65%	T-30%	T-7%	Idle mean (n=3)	std
**Genotoxic potential (*) [c)]**		ng TEQ/kg fuel	1283	1054	**886**	**1464**	**1186**	**8763**	**41288**	**2369**
Naphthalene * (**1**)	0.001	mg/kg fuel	0.006	0.005	**0.008**	**0.046**	**0.038**	**7.090**	**38.695**	1.571
Acenaphthylene (**2**)		mg/kg fuel	0.000	0.000	**0.000**	**0.001**	**0.001**	**0.125**	**0.869**	0.163
Acenaphthene (**3**)		mg/kg fuel	0.001	0.001	**0.000**	**0.001**	**0.001**	**0.039**	**0.231**	0.062
Fluorene (**4**)		mg/kg fuel	0.003	0.001	**0.002**	**0.003**	**0.003**	**0.128**	**0.649**	0.130
Phenanthrene (**5**)		mg/kg fuel	0.024	0.007	**0.029**	**0.024**	**0.023**	**0.145**	**0.551**	0.101
Anthracene (**6**)		mg/kg fuel	0.0004	0.0003	**0.0005**	**0.0004**	**0.0001**	**0.002**	**0.022**	0.009
Fluoranthene (**7**)		mg/kg fuel	0.010	0.005	**0.013**	**0.007**	**0.005**	**0.031**	**0.161**	0.029
Pyrene (**8**)		mg/kg fuel	0.013	0.006	**0.006**	**0.012**	**0.002**	**0.016**	**0.196**	0.043
Benzo(a)anthracene * (**9**)	0.1	µg/kg fuel	0.28	0.08	**0.38**	**0.25**	**0.32**	**0.46**	**3.49**	0.64
Chrysene * (**10**)	0.01	µg/kg fuel	0.74	0.37	**1.24**	**0.63**	**0.36**	**1.52**	**7.65**	0.52
Benzo(b)fluoranthene * (**11**)	0.1	µg/kg fuel	0.68	0.37	**0.56**	**0.90**	**0.47**	**0.68**	**2.00**	0.23
Benzo(k)fluoranthene * (**12**)	0.1	µg/kg fuel	0.35	0.35	**0.36**	**0.39**	**0.49**	**0.72**	**0.90**	0.39
Benzo(a)pyrene * (**13**)	1.0	µg/kg fuel	0.68	0.49	**0.36**	**0.75**	**0.48**	**0.69**	**0.91**	0.31
Dibenz(a,h)anthracene * (**14**)	1.0	µg/kg fuel	0.39	0.43	**0.34**	**0.38**	**0.49**	**0.71**	**0.83**	0.31
Benzo(g,h,i)perylene (**15**)		µg/kg fuel	1.66	1.94	**0.59**	**5.19**	**0.83**	**1.21**	**3.70**	2.59
Indeno(1,2,3-c,d)pyrene * (**16**)	0.1	µg/kg fuel	0.62	0.48	**0.35**	**1.28**	**0.50**	**0.72**	**1.33**	0.43
Sum priority PAHs (**1-16**)		mg/kg fuel	0.06	0.03	**0.06**	**0.10**	**0.08**	**7.58**	**41.40**	2.11

a) Data without background corrections
b) Sea level thrust
c) Sum of genoxtoxic PAH level multiplied by respective toxicity equivalence factor (TEF)

Table S7. Emission Indices (EI) for Priority PAHs with HEFA (32%v) and Jet A-1 Fuel (68%) Blend [a)]

	TEF	Thrust [b)]	Background mean (n=4)	std	T-85%	T-65%	T-30%	T-7%	Idle mean (n=2)	std
**Genotoxic potential (*) [c)]**		ng TEQ/kg fuel	1292	1061	**711**	**803**	**1014**	**8542**	**27160**	**933**
Naphthalene * (**1**)	0.001	mg/kg fuel	0.006	0.005	**0.008**	**0.013**	**0.013**	**6.962**	**25.847**	0.865
Acenaphthylene (**2**)		mg/kg fuel	0.000	0.000	**0.000**	**0.000**	**0.000**	**0.083**	**0.296**	0.020
Acenaphthene (**3**)		mg/kg fuel	0.001	0.001	**0.000**	**0.001**	**0.001**	**0.024**	**0.164**	0.077
Fluorene (**4**)		mg/kg fuel	0.003	0.001	**0.001**	**0.002**	**0.002**	**0.066**	**0.341**	0.153
Phenanthrene (**5**)		mg/kg fuel	0.025	0.007	**0.013**	**0.024**	**0.024**	**0.108**	**0.290**	0.085
Anthracene (**6**)		mg/kg fuel	0.0004	0.0003	**0.0002**	**0.0002**	**0.0003**	**0.001**	**0.015**	0.005
Fluoranthene (**7**)		mg/kg fuel	0.010	0.005	**0.004**	**0.009**	**0.005**	**0.033**	**0.045**	0.037
Pyrene (**8**)		mg/kg fuel	0.013	0.006	**0.002**	**0.007**	**0.002**	**0.047**	**0.044**	0.043
Benzo(a)anthracene * (**9**)	0.1	μg/kg fuel	0.29	0.08	**0.19**	**0.22**	**0.28**	**0.40**	**0.54**	0.19
Chrysene * (**10**)	0.01	μg/kg fuel	0.75	0.37	**0.62**	**0.39**	**0.26**	**1.40**	**2.66**	2.33
Benzo(b)fluoranthene * (**11**)	0.1	μg/kg fuel	0.68	0.37	**0.37**	**0.35**	**0.45**	**0.65**	**0.72**	0.16
Benzo(k)fluoranthene * (**12**)	0.1	μg/kg fuel	0.35	0.35	**0.28**	**0.32**	**0.41**	**0.59**	**0.51**	0.00
Benzo(a)pyrene * (**13**)	1.0	μg/kg fuel	0.69	0.50	**0.27**	**0.31**	**0.40**	**0.70**	**0.50**	0.00
Dibenz(a,h)anthracene * (**14**)	1.0	μg/kg fuel	0.40	0.43	**0.31**	**0.35**	**0.45**	**0.65**	**0.56**	0.00
Benzo(g,h,i)perylene (**15**)		μg/kg fuel	1.68	1.96	**0.37**	**0.65**	**0.53**	**2.91**	**0.74**	0.08
Indeno(1,2,3-c,d)pyrene * (**16**)	0.1	μg/kg fuel	0.63	0.48	**0.25**	**0.35**	**0.36**	**0.52**	**0.49**	0.05
Sum priority PAHs (**1-16**)		mg/kg fuel	0.06	0.03	**0.03**	**0.06**	**0.05**	**7.33**	**27.05**	1.29

a) Data without background corrections

b) Sea level thrust

c) Sum of genoxtoxic PAH level multiplied by respective toxicity equivalence factor (TEF)

Table S8. Fuel Effects (χ) for priority PAHs [a,b)]

	TEF	Thrust [c)]	T-85%	T-65%	T-30%	T-7%	Idle mean (n=2)	std
**Genotoxic potential (*)** [d)]		(-)	**0.80**	**0.55**	**0.86**	**0.97**	**0.66**	
Naphthalene * (**1**)	0.001	(-)	**1.02**	**0.28**	**0.33**	**0.98**	**0.67**	0.05
Acenaphthylene (**2**)		(-)		**0.10**	**0.12**	**0.66**	**0.34**	0.09
Acenaphthene (**3**)		(-)	**0.87**	**1.54**	**0.64**	**0.62**	**0.71**	0.52
Fluorene (**4**)		(-)	**0.83**	**0.88**	**0.58**	**0.52**	**0.52**	0.34
Phenanthrene (**5**)		(-)	**0.45**	**0.99**	**1.02**	**0.74**	**0.53**	0.25
Anthracene (**6**)		(-)	**0.47**	**0.52**	**1.86**	**0.41**	**0.70**	0.49
Fluoranthene (**7**)		(-)	**0.31**	**1.32**	**0.90**	**1.09**	**0.28**	0.28
Pyrene (**8**)		(-)	**0.36**	**0.58**	**0.99**	*2.9* [e)]	**0.23**	0.27
Benzo(a)anthracene * (**9**)	0.1	(-)	**0.51**	**0.88**	**0.88**	**0.87**	**0.16**	0.08
Chrysene * (**10**)	0.01	(-)	**0.50**	**0.62**	**0.73**	**0.92**	**0.35**	0.33
Benzo(b)fluoranthene * (**11**)	0.1	(-)	**0.67**	**0.39**	**0.95**	**0.95**	**0.36**	0.12
Benzo(k)fluoranthene * (**12**)	0.1	(-)	**0.78**	**0.83**	**0.83**	**0.82**	**0.57**	0.25
Benzo(a)pyrene * (**13**)	1.0	(-)	**0.77**	**0.41**	**0.83**	**1.02**	**0.54**	0.19
Dibenz(a,h)anthracene * (**14**)	1.0	(-)	**0.91**	**0.92**	**0.92**	**0.91**	**0.68**	0.26
Benzo(g,h,i)perylene (**15**)		(-)	**0.62**	**0.13**	**0.63**	*2.4* [e)]	**0.20**	0.16
Indeno(1,2,3-c,d)pyrene * (**16**)	0.1	(-)	**0.71**	**0.27**	**0.72**	**0.72**	**0.37**	0.16
Sum priority PAHs (**1-16**)		(-)	**0.67**	**0.60**	**0.83**	**0.89**	**0.45**	

a) Dimensionless ratio of $EI_{HEFA\text{-}blend}$ / $EI_{Jet\ A\text{-}1\ fuel}$
b) Data without background corrections
c) Sea level thrust
d) Sum of genoxtoxic PAH level multiplied by respective toxicity equivalence factor (TEF)
e) Considered as an outlyer
